# Multidimensional treatment foster care for preschoolers: early findings of an implementation in the Netherlands

**DOI:** 10.1186/1753-2000-6-38

**Published:** 2012-12-05

**Authors:** Caroline S Jonkman, Eva A Bolle, Robert Lindeboom, Carlo Schuengel, Mirjam Oosterman, Frits Boer, Ramon JL Lindauer

**Affiliations:** 1 Department of Child and Adolescents Psychiatry, Academic Medical Center, University of Amsterdam, Amsterdam, The Netherlands; 2 De Bascule, Academic Center for Child and Adolescents Psychiatry, Amsterdam, The Netherlands; 3 Division of Clinical Methods and Public Health, Academic Medical Center, University of Amsterdam, Amsterdam, The Netherlands; 4 Department of Clinical Child and Family Studies and the EMGO Institute for Health and Care Research, VU University, Amsterdam, The Netherlands

**Keywords:** Foster care, Preschool aged children, Behavioral problems, Attachment disturbances, Intervention

## Abstract

**Abstract:**

Multidimensional Treatment Foster Care (MTFC) has been shown to be an evidence based alternative to residential rearing and an effective method to improve behavior and attachment of foster children in the US. This preliminary study investigated an application of MTFC for preschoolers (MTFC-P) in the Netherlands focusing on behavioral outcomes in course of the intervention. To examine the following hypothesis: “the time in the MTFC-P intervention predicts a decline in problem behavior”, as this is the desired outcome for children assigned to MTFC-P, we assessed the daily occurrence of 38 problem behaviors via telephone interviews. Repeated measures revealed significant reduced problem behavior in course of the program. MTFC-P promises to be a treatment model suitable for high-risk foster children, that is transferable across centres and countries.

**Trial registration:**

Netherlands Trial Register: 1747.

## Background

Children placed in foster care have often been subject to serious maltreatment and neglect (Kohl, Edleson, English, & Barth [1]; Oswald, Heil & Goldbeck [2]). Although placement in foster care usually protects them against further exposure to child maltreatment, children have often been psychologically scarred by these experiences and as a consequence show behavioral problems (Minnis, Everett, Pelosi, Dunn & Knapp [3], Pears, Kim & Fisher [4]) and attachment problems (Smyke, Dumitrescu & Zeanah [5]; Zeanah, Scheeringa, Boris, Hellers, Smyke, & Trapani [6]). Placement in foster care most often implies that children are separated from the biological parent, which may evoke negative reactions as well. All this jeopardizes the success of foster care placements and placement failure may start a vicious circle in which the chance of another failure increases with every breakdown (Rubin, O’Reilly, Luan & Localio [7]; Oosterman, Schuengel, Slot, Bullens & Doreleijers [8]). The final option, institutional placement, is wrought with its own risk for pathological outcomes, e.g. compared to children in foster care institutionalized children show more cognitive delays (Nelson, Zeanah, Fox, Marshall, Smyke & Guthrie [9]), attachment disturbances (Smyke, Zeanah, Gleason, Drury, Fox, Nelson, Guthrie [10]) and developmental delays (Curtis, Alexander & Lunghofer [11]). To stop this vicious circle, these children and their foster parents need intensive support (Chamberlain, Price, Reid, Landsverk, Fisher & Stoolmiller [12]). Especially children with very severe behavioral problems are in need of spezialized foster care interventions [13]. These children are at high risk for placement instability (Aarons, James, Monn, Raghavan, Wells & Leslie [14]), because they have problems that may be too taxing for regular foster parents. To help foster parents provide these high-risk children with the positive and stimulating setting they need, foster parents need to learn effective behavioral management strategies and learn to provide emotional support (Fisher, Burraston & Pears [15]). To address these needs, a multidimensional treatment program for preschool foster children has been designed Chamberlain & Fisher [16].

### Multidimensional treatment foster care for preschoolers

Multidimensional Treatment Foster Care for Preschoolers (MTFC-P) combines foster care placement with evidence-based treatment of behavioral problems. Foster parents are taught effective strategies to promote positive behavior and effective limit setting for problem behavior. Concurrently children receive individually tailored behavioral interventions, focusing on problem-solving skills and prosocial behavior. Although MTFC-P is quite successful in the U.S. (see Table 1) and transportability of the MTFC model for older children has been shown in Swedish context (Westermark, Hansson and Olssen [17]), the efficacy of the preschool version has not been replicated in other countries where implementation challenges and cultural differences may play a role. The implementation of (MTFC-P) in the Netherlands offers an opportunity for such a replication.

**Table 1:** Review of publications towards MTFC-P

**Author**	**Country [year]**	**Age**	**Study Interval**	**Relative to children in regular foster care,**
				**MTFC-P children had**
Fisher, Burraston & Pears	US [2005]	3-6 years	24 months	fewer placement
Fisher, Stoolmiller, Gunnar & Burraston,	US [2007]	3-6 years	12 months	more normalized diurnal cortisol segregation
Fisher & Kim	US [2007]	3-6 years	12 months	less resistant behavior increased secure attachment
Fisher, Kim & Pears	US [2009]	3-5 years	12 months	more successful permanency attempts

The aim of this study was to preliminary and on a small-scale assess the implementation of MTFC-P in the Netherlands and test whether children enrolled in the MTFC-P program achieve desired outcomes, i.e. less problem behavior. Therefore, we addressed the following hypothesis: “the time in the MTFC-P intervention predicts a decline in problem behavior”, as this is the desired outcome for children assigned to MTFC-P.

## Method

### Participants

The first twenty children referred to MTFC-P were enrolled in the study (11 boys and 9 girls, *M*age = 5.05 years, *SD*age = 1.09, age range: 3–7 years). Although the program adheres to an age range of 3–6, also three 7-years old children enrolled, as their delayed development suggested that the intervention would fit their needs. The sample comprised 100% native Dutch children. Ethnic background of the biological parents was: 35% Surinamese, 10% Moroccan, 10% Eastern European and 45% Native Dutch. All children (100%) had experienced one or more previous placements (*M* = 3.45, *SD* = 1.47, range = 1-6) and were currently placed in non-kinship foster families.

### Intervention

#### Implementation

In 2006, Amsterdam foster care agencies initiated a covenant ‘young children in family foster care’. Within this covenant, agencies agreed that residential placement of preschool-aged children should be prevented. At that time there were no evidence-based alternatives available for preschool-aged children with behavioral problems, hence MTFC-P was implemented. Complete implementation services are provided by TFC Consultants, Inc. (see http://mtfc.com). An important focus of these services is the treatment adherence of foreign MTFC-P staff. TFC Consultants, Inc. has set some standards that prospective MTFC-P staff has to achieve, before a team is certified and allowed to use the name Multidimensional Treatment Foster Care. The purpose of TFC Consultants, Inc. implementation services and certification is to achieve positive outcomes that are similar to the outcomes previously achieved by its developers.

#### Description of intervention

MTFC-P is an intensive behavior focused program for young foster children (3 to 6 years of age), aiming to decrease children’s problem behavior and increase social behaviors, in order to promote further placement stability. MTFC-P is a treatment for children new in foster care, reentering foster care or moving between placements, all showing many problems that put them at risk for placement instability. Children are excluded from enrollment when they have an IQ <80 or when they have severe physical or psychiatric problems. Prospective MTFC-P foster parents need to attend two-day training, have to share the treatment philosophy and be willing to closely work together with MTFC-P staff. MTFC-P is delivered through a treatment team approach. A program supervisor organizes the treatment. Children receive individual training and weekly therapeutic playgroup from a skill trainer. Therapeutic foster parents participate in weekly group meetings and receive frequent home visits and ongoing support from a foster parent consultant. A family therapist supports important members of the biological family, e.g. providing biological parents with parent management strategies and concurrently guiding parent–child visits. For nine months, children are placed in a therapeutic foster family. From developmental perspectives, the family setting is considered the primary learning environment of preschool-aged children (Fisher, Ellis & Chamberlain [18]). To stimulate pro-social behavior and diminish behavioral problems, children receive behavioral interventions that are based upon Patterson’s theory of coercion with its principles of social learning (Patterson [19]). A key notion is that behavioral problems result from enforcing negative behavior and lack of modeling of positive behavior. To tackle this, MTFC-P makes use of two principal techniques. Firstly, skills trainer and therapeutic foster parents consequently reward positive behavior. Secondly, therapist and foster parents ignore negative behavior, instead they offer an alternative or put the child on a short time-out from contact. Therapeutic foster parents are responsible for the continuity of children’s behavioral interventions. To maintain a beneficial treatment setting for children, therapeutic foster parents are encouraged to stay consistent and responsive toward the child. Therapeutic foster parents receive parental strategies to encourage positive behavior and effective non-abusive limit setting for problem behavior (Chamberlain & Reid [20]; Patterson, Reid & Dishion [21]). After the initial 9 months, children are transferred to an after care setting (permanent foster family, biological parent). Here, the skills trainer continues children’s training and (foster) parents receive parenting practices to reinforce positive behavior for approximately 3 months. The children’s transfer to the permanent aftercare setting is facilitated by cooperation’s of foster care services surrounding the child, to preserve positive outcomes (Besier, Fegert, Goldbeck [22]).

### Measures

#### Problem behavior

The Child Behavioral Checklist for ages 1.5 to 5 (CBCL1.5-5; Achenbach & Rescorla [23]) and 6 to 18 (CBCL 6–18; Achenbach [24]) were filled out by foster parents to assess emotional and behavioral problems. Foster parents were asked to rate 113 items on a three point scale (0 = not at all true, 1 = somewhat true, 2 = very true), to assess internalizing and externalizing behaviors. Prior studies regarding Dutch populations found evidence for the validity of the CBCL 1.5-5 and 6–18 (Koot, Van den Oord, Verhulst & Boomsma [25]; Verhulst [26]). With regard to the present study, internal consistency for the CBCL 1.5-5 broad band syndrome scales was .75 for internalising problems (36 items), .60 for externalising problems (24 items) and .84 for total problems (73 items). Internal consistency of the CBCL version 6–18 years was good for the broad band syndrome scales externalising problems (28 items, .84) and total problems (77 items, .78). Internal consistency for internalising problems was low (32 items, .36).

#### Attachment disturbances

The Disturbance of Attachment Interview (DAI: Smyke & Zeanah [27]) is used to assess symptoms of the Reactive Attachment Disorder (RAD; Diagnostic and Statistical Manual of Mental Disorders 4^th^ edition – text revision [28]). Eight items of the DAI indicate symptoms of inhibited (5 items) or disinhibited attachment (3 items). Items are coded 0 if the symptom is definitely not present, 1 if there is some evidence for the symptom and 2 if the symptom is definitely present (Oosterman & Schuengel [8]). Criteria for a RAD classification is a score of 2 (symptom definitely present) on one of the items of the subscales. Oosterman & Schuengel [8] have suggested to exclude item 4 (‘responds reciprocally with familiar caregivers’), due to insufficiently loading on any of the DAI subscales. Two trained interviewers administer the interview to one of the foster parents, the interview is then double coded. Intraclass correlation for single measure (2-way random effects) was estimated based on the degree of agreement between the two interviewers, for the subscale Inhibition (ICC[95%] = .83), Disinhibition (ICC[95%] = .86) and Secure Base Distortion (ICC[95%] = .79). Previous research has revealed acceptable validity, internal consistency and satisfactory interrater’s reliability (Smyke, Dumitrescu & Zeanah [5]; Zeanah, Scheeringa, Boris, Heller, Smyke & Trapani [6]).

#### Daily problem behavior during MTFC-P

The Parent Daily Report (PDR; Chamberlain & Reid [20]) is a telephone interview with one of the foster parents and is conducted daily during weekdays, to assess the presence of 38 problem behaviors (e.g. cruelty to animals, arguing) within the past 24 hours that we scored at a two-point scale (0 = not occurred, 1 = occurred at least once). The PDR has been used as a measure for treatment outcomes previously and psychometric properties have been found adequate (Chamberlain, Price, Reid, Landsverk, Fisher & Stoolmiller [12]).

#### Procedures

A Medical Ethical Committee approved the study. Assessment of behavioral problems was scheduled one month after placement because children were placed in new foster families when entering the program. A new foster setting is often accompanied by a temporary decrease or increase of problems. The DAI was scheduled within the third month after children entered their new foster family, assuming this is a plausible period for the development of an attachment relation between child and foster parent (Stoval & Dozier [29]). Child maltreatment was registered based on records from child protective services at the end of the treatment. To examine the development of behavioral problems over the course of the intervention, a trained caller administered the PDR, to the MTFC-P foster parents daily by telephone at weekdays. Because the development of problem behavior was assessed in an open and uncontrolled way, careful interpretation of the results is needed.

### Statistical analysis

Analyses were done with SPSS version 17.0. We analyzed the relationship between problem behavior and time in intervention using a linear mixed model.

## Results

Results revealed that a large proportion of MTFC-P children had been exposed to different forms of child maltreatment. Furthermore, foster parents reported high incidence of symptoms of attachment disorder and increased levels of problem behavior (see Table 2).

**Table 2:** Child maltreatment, symptoms of attachment disorder and problem behavior

	**% (*n*)**	
***Child Maltreatment***		
Physical Abuse	42 (8)	
Sexual Abuse	10 (2)	
Neglect	95 (19)	
***Symptoms of Disturbance of Attachment***		
Inhibition	31 (5)	
Disinhibition	44 (7)	
RAD	50 (8)	
***Problem Behavior***		
	M (SD)	Cut off %
Internal	61.56 (11.59)	43.8
External	59.13 (12.09)	31.3
Total	62.31 (13.45)	50.0

**Figure 1:**
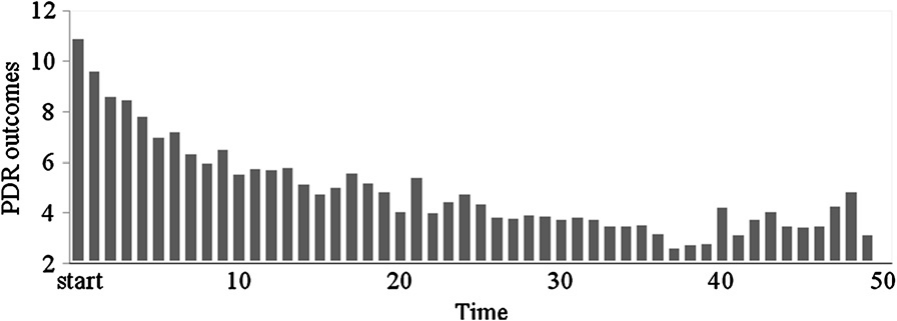
Problem behavior (frequencies) by time (weeks).

With regard to daily problem behavior, foster parents reported a fitted mean of 8.77 (*SE* = .69) per week at baseline. Frequencies of problem behavior decreased over time (Figure 1) from a daily mean of 10.99 (*SD* = 7.58) in the first week to a daily mean of 3.21(*SD* = 2.16) in the fiftieth week. Fixed effects demonstrated that the variable ‘time’ was a strong predictor of PDR outcomes (*p* < = .001, *95% CI* = −0.18 to −0.08) and indicated a mean 0.13 (*SE* = .02) lower occurrence of reported problem behaviors per week: approximately one problem behavior less every eight weeks (1/0.13 = 8).

## Discussion

This preliminary study of MTFC-P in a Dutch sample of twenty children demonstrated that time in the intervention predicts a decline in problem behavior. Behavioral problems reported by the foster parents gradually diminished during the intervention.

Our small sample size does not allow us to judge whether this is typical for children in the Netherlands referred for MTFC-P. This will become clear from our larger study of MTFC-P that is currently carried out. Because of the relatively small sample size and because the study is uncontrolled, we have to be careful in interpreting the decline of problems during the MTFC-P as resulting from the intervention, rather than (for instance) passage of time, or getting used to the foster family. Our study was further limited in that we only used self-reports of therapeutic foster parents on a single measure, the PDR. However, we suggested that the therapeutic foster parents would be the most reliable coders for problem behavior as they operate as semi-professionals and are best aware of children’s behavior. Furthermore, we choose the PDR, as this daily assessment of problem behavior is least biased by time of recall. The use of multi-informant (Lanktree [30]) and multi-method assessment (e.g. observations, physiological measures) would have been advisable, but these limitations are according to the typical characteristics of a pilot study. Nevertheless, these are promising results, consistent with findings in more rigorous studies of MTFC-P showing that, relative to children in regular foster care, children in MTFC had less resistant behavior [31] and at the end of MTFC-P children had more desired outcomes.

## Conclusions

Notwithstanding these limitations, our study was able to demonstrate that MTFC-P is a promising intervention when provided to a group of children with severe problem behavior and attachment disturbances in the Netherlands. Nonetheless, further studies towards MTFC-P are recommended to include a randomized and controlled research design to examine generalizability of treatment outcomes. The present study is a small step towards more knowledge about treatment of young foster children and a promising intervention for young foster children with severe behavioral problems.

## Competing interests

The authors declare that they have no competing interests.

## Authors’ contribution

Recruitment of participants, data gathering and data analyses are executed by C.S. Jonkman and E.A. Bolle and coordinated by C.S. Jonkman. All other authors participated in the planning, supervision and co-ordination of the study. C.S. Jonkman wrote the manuscript, in cooperation with the other authors. All authors have critically read and approved the submitted manuscript.

## Authors’ information

*Caroline S. Jonkman, MSc.* Is child psychologist and PhD student at the department of Child and Adolescent Psychiatry at the AMC-Academic Medical Center (University of Amsterdam, the Netherlands).

*Eva Bolle, MSc.* Is child psychologist and research assistant at the department of therapeutic foster care of the academic center for Child and Adolescent Psychiatry De Bascule (Amsterdam, The Netherlands).

*Prof. Dr Carlo Schuengel* Is professor at VU University and EMGO institute for Health and Care Research and head of the department of Clinical Child and Family Studies and Special Education (Amsterdam, the Netherlands).

*Dr. Robert Lindeboom* Is clinical epidemiologist at the department of Clinical Epidemiology and Biostatistics at the AMC-Academic Medical Center (University of Amsterdam, the Netherlands).

*Dr. Mirjam Oosterman* Is assistant professor at VU University and EMGO institute for Health and Care Research and head of the department of Clinical Child and Family Studies and Special Education (Amsterdam, the Netherlands).

*Prof. Dr. Frits Boer* Is emeritus professor of the department of Child and Adolescent Psychiatry at the AMC-Academic Medical Center (University of Amsterdam, the Netherlands).

*Dr. Ramón J.L. Lindauer* Is child and adolescent psychiatrist and family therapist and head of the department of Child and Adolescent Psychiatry at the AMC-Academic Medical Center (University of Amsterdam, the Netherlands).
